# Association between Fractional Oxygen Extraction from Resting Quadriceps Muscle and Body Composition in Healthy Men

**DOI:** 10.3390/jfmk8040149

**Published:** 2023-10-26

**Authors:** Rodrigo Yáñez-Sepúlveda, Jorge Olivares-Arancibia, Guillermo Cortés-Roco, Aldo Vasquez-Bonilla, Matías Monsalves-Álvarez, Ildefonso Alvear-Órdenes, Marcelo Tuesta

**Affiliations:** 1 Faculty Education and Social Sciences, Universidad Andres Bello, Viña del Mar 2520000, Chile; rodrigo.yanez.s@unab.cl; 2 Grupo AFySE, Investigación en Actividad Física y Salud Escolar, Escuela de Pedagogía en Educación Física, Facultad de Educación, Universidad de las Américas, Santiago 8320000, Chile; 3 Faculty Education, Universidad Viña del Mar, Viña del Mar 2520000, Chile; guillermo.cortes@uvm.cl; 4 Faculty of Sport Sciences, Universidad de Extremadura, 10001 Caceres, Spain; alvasquezb@unex.es; 5 Instituto de Ciencias de la Salud, Universidad de O’Higgins, Rancagua 2820000, Chile; matias.monsalves@uoh.cl; 6 Applied Physiology Laboratory (FISAP), Institute of Biomedicine (IBIOMED), University of León, 24001 León, Spain; ialvor@unileon.es; 7 Exercise and Rehabilitation Sciences Laboratory, School of Physical Therapy, Faculty of Rehabilitation Sciences, Universidad Andres Bello, Viña del Mar 2520000, Chile; marcelo.tuesta@unab.cl; 8 Laboratory of Sport Sciences, Centro de Medicina Deportiva Sports MD, Viña del Mar 2521156, Chile

**Keywords:** spectroscopy, near-infrared, body composition, health, cell respiration

## Abstract

This study aimed to associate body composition with fractional oxygen extraction at rest in healthy adult men. Fourteen healthy adults (26.93 ± 2.49 years) from Chile participated. Body composition was assessed with octopole bioimpedance, and resting muscle oxygenation was evaluated in the vastus lateralis quadriceps with near-infrared spectroscopy (NIRS) during a vascular occlusion test, analyzing the muscleVO_2_, resaturation velocity during reactive hyperemia via the muscle saturation index (%TSI), and the area above the curve of HHb (AACrep). It was observed that the total and segmented fat mass are associated with lower reoxygenation velocities during hyperemia (*p* = 0.008; β = 0.678: *p* = 0.002; β = 0.751), and that the total and segmented skeletal muscle mass are associated with higher reoxygenation velocities during hyperemia (*p* = 0.020; β = −0.614: *p* = 0.027; β = −0.587). It was also observed that the total and segmented fat mass were associated with a higher area above the curve of HHb (AACrep) during hyperemia (*p* = 0.007; β = 0.692: *p* = 0.037; β = 0.564), and that total and segmented skeletal muscle mass was associated with a lower area above the curve of HHb (AACrep) during hyperemia (*p* = 0.007; β = −0.703: *p* = 0.017; β = −0.632). We concluded that fat mass is associated with lower resaturation rates and lower resting fractional O_2_ extraction levels. In contrast, skeletal muscle mass is associated with higher resaturation rates and fractional O_2_ extraction during reactive hyperemia. The AACrep may be relevant in the evaluation of vascular adaptations to exercise and metabolic health.

## 1. Introduction

Physical inactivity and obesity have been linked to an increased prevalence of vascular diseases [1,2], In recent years, endothelial dysfunction (ED) has increased in the population [3]. ED is defined as an abnormality of the vascular system that serves as a predictor of cardiovascular events [4], Consequently, monitoring vascular function has gained significant importance in the prevention of vascular events [5].

Various techniques are used to assess endothelial dysfunction. Near-infrared (NIRS) has emerged as a valuable tool for monitoring muscle metabolism, fractional O_2_ extraction, and muscle resaturation velocity. This compact, non-invasive, wireless, and low-cost technology enables the real-time analysis of vascular parameters and provides insights into fractional O_2_ extraction [6,7,8]. Fractional O_2_ extraction, which represents the dynamic balance between oxygen utilization and supply, offers crucial information regarding fundamental vascular physiological mechanisms. This information has applications in both sports and clinical settings, allowing for the analysis of the effects of physical exercise on muscle oxygenation, hemodynamics, and metabolism [9,10]. NIRS is widely utilized during exercise and sports research, with most studies relying on the tissue saturation index (TSI) to describe fractional O_2_ extraction [11].

Moreover, it has been observed that NIRS can be utilized to assess muscle oxygen consumption at rest using the arterial occlusion technique. Following the blockage of blood flow, a reactive hyperemia response ensues, characterized by increased blood flow to the affected area compared to the normal resting blood flow. This enables the analysis of microvascular reactivity and muscle microvascular vasodilatory function [12], providing data on the rate of muscle reoxygenation [13]. The reactive hyperemia represents a response to ischemia induced by arterial occlusion, where the downstream microvasculature exhibits relaxation of the vascular smooth muscle. This leads to a rapid and exaggerated increase in arteriolar blood flow upon removal of the occlusion [14]. The rate of resaturation during hyperemia can be analyzed and delivers valuable data on peripheral vascular function [12]. Consequently, reactive hyperemia is a valid technique for the noninvasive assessment of peripheral microvascular function, as well as a powerful predictor of all-cause cardiovascular morbidity and mortality [15].

On the clinical side, it is important to note that the microvascular reactive hyperemic response of the muscle following occlusion tends to be reduced with vascular-impaired conditions, such as insulin resistance and obesity [16]. Lifestyle choices can significantly influence this hyperemic response. For example, individuals who regularly engage in cycling and running exercises exhibit greater microvascular responses in their lower extremities compared to sedentary individuals [17]. While previous studies have compared muscle oxygenation profiles in physically active and inactive subjects, limited evidence exists regarding the relationship between body composition and resaturation velocity during reactive hyperemia in healthy men. Hence, the measurement of local hemodynamics in skeletal muscle has the potential to provide valuable information on oxygen supply to tissues, both during exercise and at rest [18]. Their relevance is underscored by recent research demonstrating a connection between microvascular responses to exercise [14]. Previous studies have reported that improved physical performance can induce beneficial changes in the muscular microvascular response. These improvements can be attributed to increased endothelial response [19], angiogenesis [20], a heightened increase in mitochondrial density and oxidative capacity [21], reduced levels of shear stress, lower blood pressure, and improved vasodilation [22], all of which favor greater fractional oxygen extraction [9]. Body composition influences vascular function; higher levels of body fat lead to increased production of adipokines and proinflammatory cytokines, ultimately resulting in endothelial dysfunction [23]. Conversely, higher levels of muscle mass are associated with improved endothelial function [24]. While it has been reported that the percentage of skeletal muscle mass is a strong predictor of cardiorespiratory fitness [25], there is limited evidence connecting muscle resaturation during reactive hyperemia to body composition in healthy subjects.

Based on the previous literature, we hypothesized that body fat and muscle mass would be associated with microvascular resaturation at the muscle level during reactive hyperemia when analyzed with NIRS. Finally, the study aimed to establish an association between body composition and fractional oxygen extraction at rest in healthy adult men.

## 2. Materials and Methods

### 2.1. Ethical Considerations

The study was approved by the scientific ethics committee of Universidad Viña del Mar under code R19-02. The research was performed following the recommendations of the Helsinki Declaration for human studies [26].

### 2.2. Participants

Eighteen healthy men from the Valparaíso region of Chile were included. The inclusion criteria required participants to have no clinical conditions and a vastus lateralis quadriceps skinfold in the area of NIRS location < 16 mm. They were also required to provide informed consent by means of a signature. The exclusion criteria encompassed the use of tobacco or recreational drugs, the use of vasodilator medications or foods, and non-participation in all evaluation sessions.

### 2.3. Evaluations

The evaluations were performed in the clinical laboratory of a hospital. The ambient temperature of the site was between 18° and 22° Celsius. Trained professionals who were blinded to the study’s purpose conducted the evaluations.

### 2.4. Body Composition

Body composition was assessed using an INBODY^®^ model 270 (Seoul, Republic of Korea) octopolar bioimpedance meter, which had previously been validated for this population [27]. Three assessments were conducted, and the median result was used for the study. The values of fat mass (%) and muscle mass (%), segmented muscle mass (SMM), and segmented fat mass (SFM) in kilograms were used for the analysis.

### 2.5. Muscle Resaturation with NIRS

Before the evaluation, participants were instructed to abstain from consuming caffeine or stimulants, avoid foods with high nitrate levels for at least 8 h before the evaluations, refrain from exercise for 48 h before the measurement, and ensure that they had a sleep duration ranging from 8 to 9 h on the night before the assessment. Participants received prior training on minimizing movement during the evaluation.

**Figure 1:**
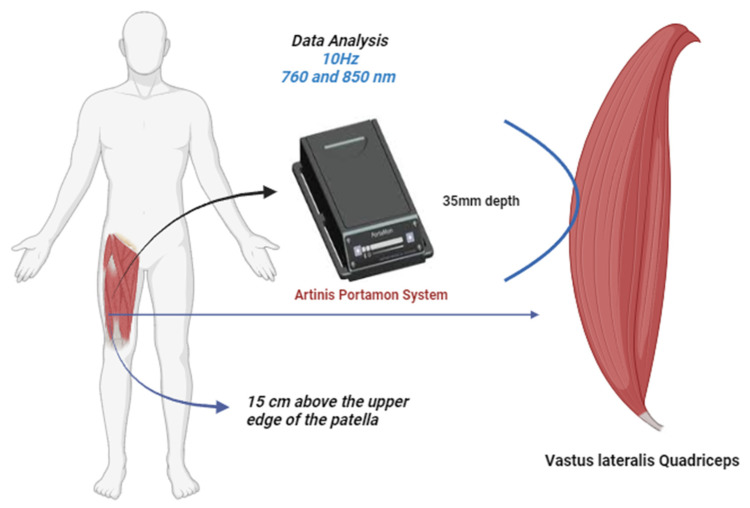
Evaluation protocol.

Once in the laboratory, the evaluations were performed after 10 min of rest in the supine position on a clinical couch. Heart rate and blood pressure were measured using an OMRON^®^ model HEM7600 monitor (Kyoto, Japan). Before the evaluation, the measurement area was shaved, and the thigh crease was measured using a Harpenden^®^ adipometer (Hertfordshire, UK). The vascular occlusion test was applied to the right vastus lateralis quadriceps, with the cuff placed in the most proximal area of the assessed limb and set at a pressure of 240–260 mmHg [28]. The occlusion was maintained for 5 min, and reactive hyperemia was analyzed for 8 min. Muscle resaturation was assessed using a continuous wave (CW) device, specifically the Portamon model by Artinis Medical Systems^®^, (Einsteinweg, Netherlands). This device was positioned on the distal part of the right vastus lateralis muscle (15 cm above the patella’s upper edge) in the direction of the muscle fibers. The device was programmed to operate at a sampling frequency of 10 Hz, utilizing wavelengths of 760 and 850 nm [29]. To prevent interference from ambient light, a black cloth was placed over the device during the evaluation (refer to Figure 1).

### 2.6. Muscle VO_2_ and the Area above the Curve of HHb

Muscle VO_2_ was assessed using arterial occlusion, taking into account the rate of decrease in O_2_Hb, assuming that the total hemoglobin (tHb) remained constant. The rate of increase in concentrations was used to calculate the absolute muscle VO_2_ value once the venous outflow had been blocked. The data were converted from micromolars per second (mM/s) to milliliters of O_2_ per 100 g of tissue per minute (mL/100 g/min) [30].

The area above the curve of HHb (AACrep), which represents the reperfusion period, was calculated as the total area above the curve from cuff release to 8 min after reperfusion [31].

### 2.7. Data Analysis

O_2_Hb and HHb were determined using the third channel signals of the Portamon device (35 mm tissue penetration), the Portamon Oxysoft (Einsteinweg, Netherlands) program, and the application of the modified Lambert–Beer law. The TSI was calculated using the following formula: %TSI = [O_2_Hb]/([O_2_Hb] + [HHb]) × 100 [32]. The baseline TSI (%) was calculated 2 min before applying the occlusion, once the NIRS signal had stabilized. The TSI reperfusion rate was analyzed in two ways: (1) the time from the trough during occlusion to the baseline during reactive hyperemia; and (2) the time from the trough during occlusion to the peak reported during hyperemia.

### 2.8. Statistical Analysis

The mean, standard deviation, and 95% confidence intervals were used to describe the study variables. Then, a Shapiro–Wilk test was applied, observing a normal data distribution (*p* < 0.05). Subsequently, a Pearson correlation test was applied to estimate whether the variables were associated. The magnitude of the correlation effect was based on the following scale: trivial (<0.10), small (0.10–0.29), moderate (0.30–0.49), high (0.50–0.69), very high (0.70–0.89), almost perfect (≥0.90), and perfect (r = 1.00) [33]. Then, two linear regression models were performed (model 1: % body fat; model 2: % muscle mass) and associated with the resaturation velocity during reactive hyperemia, where the beta coefficient (β) was used. All linear regressions were adjusted for BMI. For all analyses, *p* < 0.05 was considered statistically significant. Statistical analysis was done using Jamovi software (Sydney, Australia) version 2.3.21.

## 3. Results

Table 1 shows the study variables and confidence intervals. Our target population consisted of healthy young men with a mean age of 26.93 ± 2.49. A minimum to maximum post-occlusion time resaturation velocity of 18.54 s and a minimum to baseline post-occlusion resaturation value (s) of 8.31 s were obtained.

**Figure 2:**
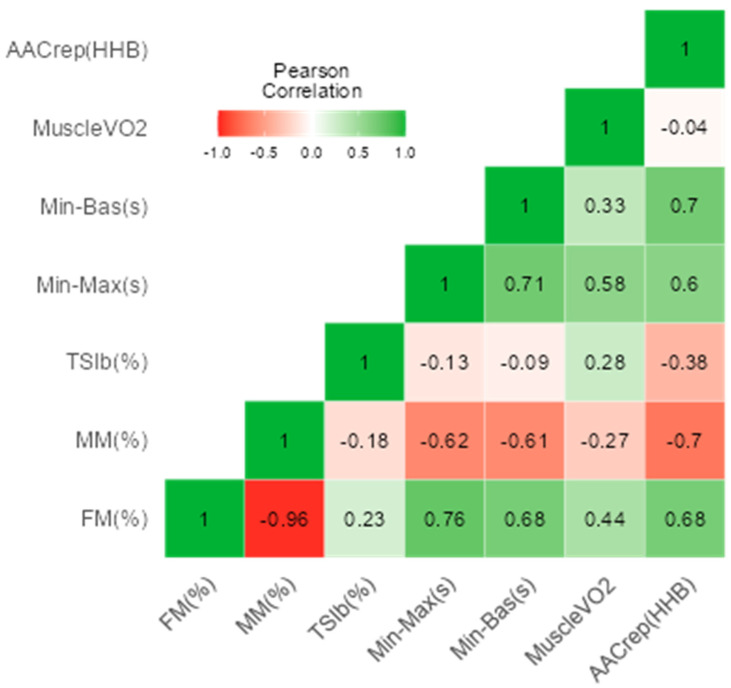
Correlation matrix of body composition and muscle resaturation variables. FM: fat mass; AACrep (HHb): Area above the curve of the NIRS-derived deoxyhemoglobin: MM: muscle mass; Min-Max: minimum to maximum post-occlusion time (s); Min-Bas: Minimum to baseline post-occlusion (s).

In Figure 2, negative correlations can be observed between muscle and fat mass (R = −0.96; *p* < 0.001). In addition, positive relationships were observed between fat mass (%) and minimum to maximum post occlusion (s) (R = 0.76; *p* = 0.001) and with minimum to baseline post occlusion (s) (R = 0.68; *p* = 0.008). Muscle mass was negatively related to minimum to maximum post-occlusion time (s) (R = −0.62; *p* = 0.018) and to minimum to baseline post-occlusion time (s) (R = −0.61; *p* = 0.020).

**Figure 3:**
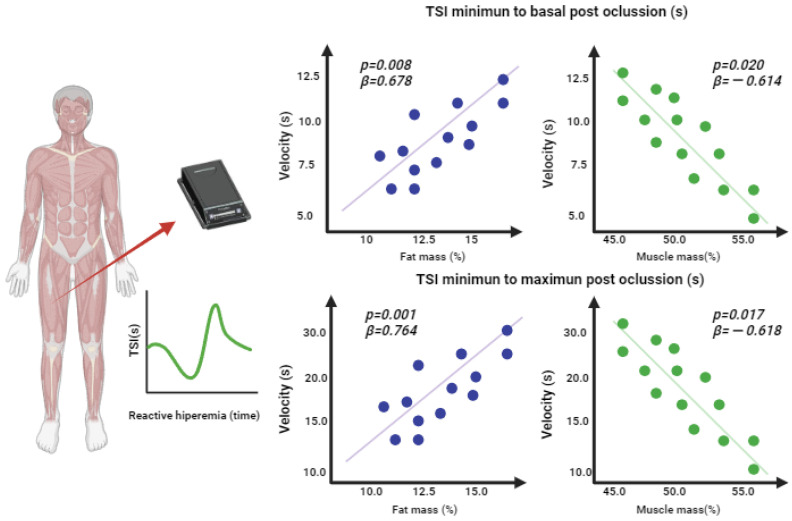
Linear regression of body composition and muscle resaturation variables during reactive hyperemia.

Figure 3 shows the *p*-values and beta indicators of the linear regressions of the components of body composition and resaturation velocity during reactive hyperemia. A lower reoxygenation velocity was observed, with a positive association between fat mass and resaturation velocity from the minimum to the basal value (*p* = 0.008; β = 0.678) and velocity from minimum to maximum (*p* = 0.001; β = 0.764). In contrast, skeletal muscle mass was associated with higher resaturation velocities during hyperemia from the minimum to the basal value (*p* = 0.020; β = −0.614) and from the minimum to the maximum value (*p* = 0.020; β = −0.618).

**Figure 4:**
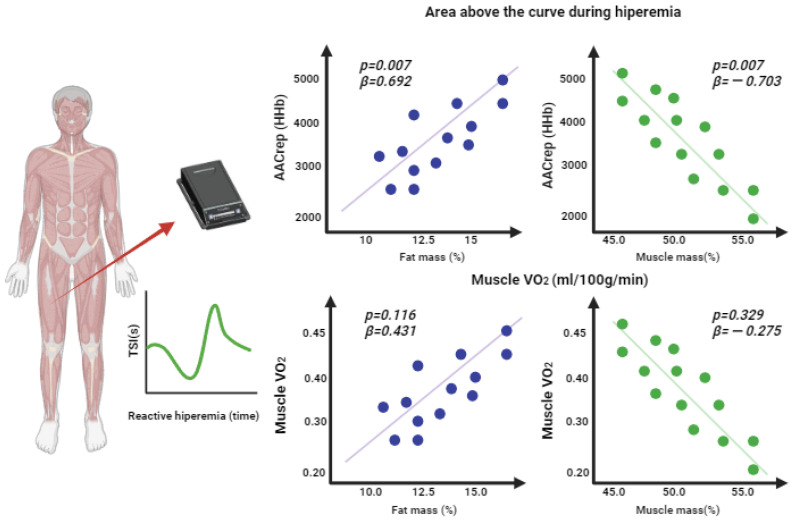
Linear regression of body composition, the area above the curve during hyperemia, and muscle VO_2_.

Figure 4 shows the *p*-values and beta indicators of the linear regressions of the components of body composition, the area above the curve during hyperemia, and muscle VO_2_. A greater area above the HHb curve was observed as the fat mass increased (*p* = 0.007; β = 0.692), and a smaller area above the HHb curve was observed as the skeletal muscle mass increased (*p* = 0.007; β = −0.703). No associations were found between fat mass and muscle VO_2_ (*p* = 0.116; β = −0.431), and muscle mass was also not associated with muscle VO_2_ (*p* = 0.329; β = −0.275).

**Figure 5:**
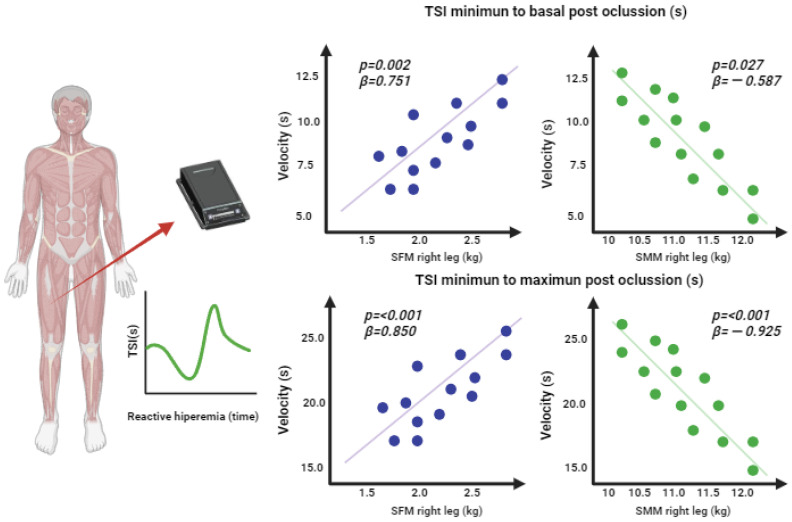
Linear regression of the segmented body composition of the right leg (kg)and muscle resaturation variables during reactive hyperemia.

Figure 5 shows the *p*-values and beta indicators of the linear regressions of the components of the segmented body composition of the right leg (kg), as well as the resaturation velocity during reactive hyperemia. A lower reoxygenation velocity was observed, with a positive association between segmented fat mass and resaturation velocity from the minimum to the basal value (*p* = 0.002; β = 0.751) and with velocity from minimum to maximum (*p* ≤ 0.001; β = 0.850). In comparison, segmented skeletal muscle mass was associated with higher resaturation velocities during hyperemia from the minimum to the basal value (*p* = 0.027; β = −0.587) and from the minimum to the maximum value (*p* ≤ 0.001; β = −0.925).

**Figure 6:**
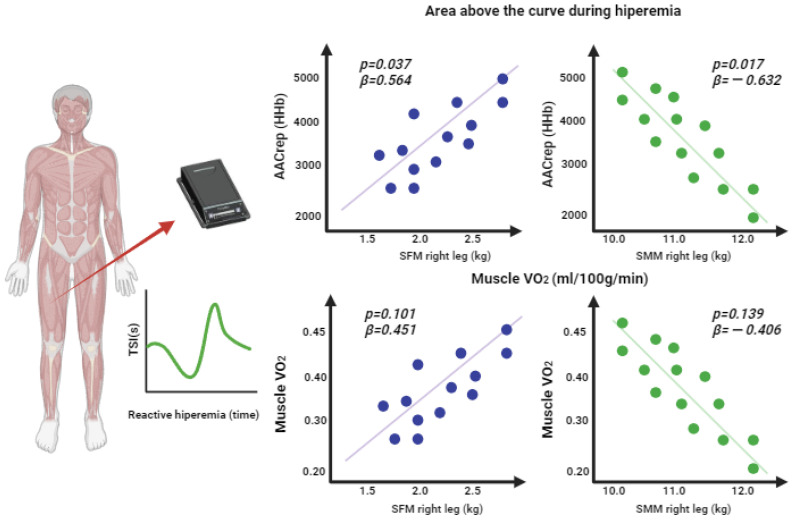
Linear regression of the segmented body composition of the right leg (kg), the area above the curve during hyperemia, and muscle VO_2_.

Figure 6 shows the *p*-values and beta indicators of the linear regressions of the components of the segmented body composition of the right leg (kg), the area above the curve during hyperemia, and muscle VO_2_. A greater area above the HHb curve was observed as the segmented fat mass increased (*p* = 0.037; β = 0.564), and a smaller area above the HHb curve was found as the segmented skeletal muscle mass increased (*p* = 0.017; β = −0.632). No associations were found between segmented fat mass and muscle VO_2_ (*p* = 0.101; β = −0.451); segmented muscle mass was not associated with muscle VO_2_ either (*p* = 0.139; β = −0.406).

## 4. Discussion

The present study aimed to associate body composition with fractional oxygen extraction at rest in healthy adult men. The study’s results indicate that a higher level of body fat was associated with lower post-occlusion resaturation velocity, and that skeletal muscle mass was associated with higher muscle resaturation velocities.

### 4.1. Hyperemia Reactive Response

Studies have indicated that mean resaturation time during reactive hyperemia represents a microvascular measure of resting muscle, which is associated with functional health in young adults [14]. The increased resaturation rate occurs because of a muscle fractional extraction of O_2_ after a period of O_2_ shortage, for example, during ischemia of arterial occlusion at rest. The improvements in the fractional O_2_ extraction are due to a more significant dilation of the arterioles, which reduces vascular resistance and, consequently, a greater blood flow associated with an improvement in mitochondrial density [34]. Likewise, research has shown that increased angiogenesis enhances the delivery of nutrients to myocytes during peripheral reperfusion [35], which in turn can be observed in increases in muscle mass in healthy subjects [36,37].

A study performed in hypertensive patients where microvascular reactivity was analyzed with Laser Speckle Contrast Image and pulse wave showed that higher body adiposity was associated with lower microvascular reactivity [38], similar to our study. The authors attribute the negative effects of obesity to the inflammation that vascular adipose tissue generates, increasing endothelial dysfunction by an increase in the secretion of vasoconstriction factors and proinflammatory adipokines that cause vascular inflammation and decrease endothelial activation [39]. It has also been evidenced that increased body fat is associated with lower levels of nitric oxide generation and higher levels of arterial stiffness [40], which contributes to lower muscle resaturation velocity during reactive hyperemia.

### 4.2. Relationship between Adipose and Muscle Mass with the Hyperemic Response

Possible associations between greater muscle mass and an improvement in the hyperemic response have been explained by Soares et al. (2018), who examined the microvascular response of skeletal muscle in different extremities (trained vs. untrained) and found that the vascular adaptations induced by resistance training are more prominent in the trained limb compared to the hyperemic response of the untrained limb [17]. Additionally, it is relevant to consider a recent study that reported a moderate correlation (r = 0.56) between muscle mass and muscle O_2_ resaturation following post-occlusive [41]. Other studies have reported differences in the vascular occlusion response measured by NIRS between men and women, attributing these differences to gender-specific muscular phenotypic characteristics [42], reinforcing the findings of the present study.

Recently, Jones et al. (2022) reported that NIRS-derived parameters in the gastrocnemius muscle during reactive hyperemia were associated with improved performance in muscle oxygen saturation during a step test in older adults with higher levels of adipose tissue, and this may be a determinant for future cardiovascular diseases [18]. Also, previous research has consistently shown that absolute NIRS measurements are attenuated by adipose tissue thickness [43]. Curiously, the mean resaturation time was found to be unrelated to skinfold thickness, indicating that this parameter was not affected by the limitation of this methodology due to the instrument’s (Portamon) high sensitivity in detecting local adipose tissues larger than >7 mm [44]. It’s important worth mentioning that our study did not have local measurements of the adipose tissue of the muscle evaluated. However, it may be that the local adipose tissue has little direct influence on the hyperemic response and whether the systemic fat percentage has been evaluated in this study [45].

Therefore, the present findings suggest that mean resaturation time could serve as a good measure of muscle microvascular reactivity when skinfold thickness (i.e., adipose tissue thickness) is variable among participants in a research study [14]. Vásquez-Bonilla et al. reported that increased TSI is a limiting factor of performance and that resaturation velocity, analyzed with NIRS, can be a valuable tool for evaluating muscle performance [7]. It has recently been reported that the reperfusion rate during reactive hyperemia is strongly associated with aerobic capacity (VO_2_max) and is a good indicator for analyzing the metabolic quality of muscle [46].

### 4.3. Clinical Implications

Our study found that skeletal muscle mass was associated with lower values in the area above the HHb curve during reactive hyperemia. Previous studies have shown that the HHb signal is less susceptible to changes in blood volume than the O_2_Hb signal in most cases, so using HHb would be a good indicator of fractional O_2_ extraction at rest [47]. In this context, it has been shown that a reduced area above the curve is associated with a higher fractional O_2_ extraction derived from a greater capacity to switch from aerobic to anaerobic metabolism during reactive hyperemia, as occurs in trained subjects [17,31]. Therefore, observing peripheral adaptations to exercise can be performed using HHb by NIRS.

Likewise, a study revealed that the decreased deoxyhemoglobin area over the curve during reperfusion could indicate greater metabolic flexibility that could be conditioned by higher levels of muscle mass derived from more metabolically efficient muscles [48].

Additionally, the physiological adaptations that occur in muscle oxygenation, angiogenesis, muscle mass, and maximum oxygen consumption, among other factors, can explain improved muscle metabolism at rest and during exercise [49,50].

Finally, the post-occlusive response causes a vasodilatory response that indirectly evaluates vascular endothelial function [51]. Vascular endothelial function has been a useful predictor of cardiovascular disease (CVD) risk [15]. Importantly, peripheral impairment contributes to the exercise intolerance that is observed in different clinical populations, such as those with heart failure (CHF), diabetes mellitus, and hypertension, among other diseases [39]. Therefore, determining muscle oxygen AACrep (HHb) offers a direct reflection of cardiovascular fitness levels [17], which may be useful for monitoring vascular health in clinical or sedentary populations [42,52].

### 4.4. Limitations

The limitations of this study are that the study sample was small for a cross-sectional study. Likewise, studies with control groups and interventions demonstrating AACrep (HHb) adaptions are needed. For future research, a more representative sample of the studied population is suggested, as is studying the association between body composition and peripheral fractional oxygen extraction at rest in people with different nutritional states, health conditions, and levels of physical condition. This would allow us to demonstrate associations between these variables in healthy subjects or patients.

## 5. Conclusions

It is concluded that body fat is associated with a lower rate of muscle O_2_ resaturation at rest, and that skeletal muscle mass is related to a higher rate of muscle resaturation in healthy men. Also, the arterial occlusion test with NIRS may be a factor to be considered for the analysis of peripheral vascular response and deterioration of metabolic health.

## Figures and Tables

**Table 1:** Baseline characteristics of the vascular variables and muscle resaturation time.

Variable	Mean		SD	95% CI	
				Lower	Upper
Age (years)	26.9	±	2.4	25.4	28.3
Weight (kg)	74.8	±	5.9	71.3	78.2
Height (m)	1.74	±	0.05	1.70	1.77
BMI (weight/height^2^)	24.83	±	1.74	23.83	25.83
Fat mass (%)	12.16	±	4.09	9.79	14.52
Muscle mass (%)	50.25	±	2.83	48.61	51.89
Fat mass, right leg (kg)	1.87	±	0.42	1.63	2.11
Muscle mass, right leg (kg)	11.15	±	1.15	10.48	11.82
Heart rate (bpm)	53.2	±	6.3	49.5	56.9
Systolic pressure (mmHg)	126.00	±	10.66	119.84	132.16
Diastolic pressure (mmHg)	73.93	±	7.80	69.42	78.43
TSI Baseline (%)	68.5	±	2.91	66.9	70.2
MuscleVO_2_ (mL/100 g/min)	0.30	±	0.08	0.26	0.35
Minimum to maximum post-occlusion time (s)	18.54	±	5.01	15.65	21.43
Minimum to baseline post-occlusion time (s)	8.31	±	3.08	6.53	10.09
AACrep (HHb)	3842	±	979	3276	4407

AACrep (HHb): Area above the curve of the NIRS-derived deoxyhemoglobin.

## Data Availability

The data of this study are available upon request from the corresponding author.
